# Equitable access to cancer patient pathways in Norway – a national registry-based study

**DOI:** 10.1186/s12913-021-07250-1

**Published:** 2021-11-25

**Authors:** Frank Olsen, Bjarne K. Jacobsen, Ivar Heuch, Kjell M. Tveit, Lise Balteskard

**Affiliations:** 1 Department of Community Medicine, UiT The Arctic University of Norway, Tromsø, Norway; 2 Centre for Clinical Documentation and Evaluation (SKDE), Northern Norway Regional Health Authority, Tromsø, Norway; 3 Centre for Sami Health Research, UiT The Arctic University of Norway, Tromsø, Norway; 4 Department of Mathematics, University of Bergen, Bergen, Norway; 5 Institute of Clinical Medicine, Faculty of Medicine, University of Oslo, Oslo, Norway

**Keywords:** Norway, Critical pathways, Universal health care, Cancer, Socioeconomic factors, Small-area analysis

## Abstract

**Background:**

In 2015, cancer patient pathways (CPP) were implemented in Norway to reduce unnecessary non-medical delay in the diagnostic process and start of treatment. The main aim of this study was to investigate the equality in access to CPPs for patients with either lung, colorectal, breast or prostate cancer in Norway.

**Methods:**

National population-based data on individual level from 2015 to 2017 were used to study two proportions; i) patients in CPPs without the cancer diagnosis, and ii) cancer patients included in CPPs. Logistic regression was applied to examine the associations between these proportions and place of residence (hospital referral area), age, education, income, comorbidity and travel time to hospital.

**Results:**

Age and place of residence were the two most important factors for describing the variation in proportions. For the CPP patients, inconsistent differences were found for income and education, while for the cancer patients the probability of being included in a CPP increased with income.

**Conclusions:**

The age effect can be related to both the increasing risk of cancer and increasing number of GP and hospital contacts with age. The non-systematic results for CPP patients according to income and education can be interpreted as equitable access, as opposed to the systematic differences found among cancer patients in different income groups. The inequalities between income groups among cancer patients and the inequalities based on the patients’ place of residence, for both CPP and cancer patients, are unwarranted and need to be addressed.

**Supplementary Information:**

The online version contains supplementary material available at (10.1186/s12913-021-07250-1).

## Background

Cancer patient pathways (CPP) have been established in several countries to avoid an undesirable delay in cancer diagnosis and treatment. In the early 2000s, urgent referral pathways were introduced in the UK and in Spain, targeting an upper limit of two weeks between seeing a general practitioner (GP) to being referred to a specialist at a hospital [1, 2]. Denmark implemented CPPs in 2007–2008 [3, 4], and Sweden during the years 2015-2018 [5]. In addition to reducing and standardising waiting times, in Denmark CPPs were also intended to improve survival of cancer patients.

Norway introduced CPPs in January 2015 for lung, colorectal, breast and prostate cancers [6–9]. These cancer sites represented approximately 50% of all new cases, as well as half of all cancer-related mortality in 2016 [10]. Later in 2015, another 24 CPPs were implemented. All Norwegian CPPs were based upon Norwegian guidelines for diagnosis, treatment and follow-up of the specific cancer groups [11]. The aim was to reduce unnecessary non-medical delay in the diagnostic and start of treatment period and to increase satisfaction, quality and predictiveness to patients with a suspicion of cancer [12].

Patients are referred to a CPP by a GP, a specialist in private practice or a specialist in a public hospital if the doctor has a “justified suspicion of cancer” [11]. The suspected cancer diagnosis should be based on a set of symptoms and tests, described in national guidelines for CPPs [6–9], and the referral should be labelled as “cancer patient pathway”. There are three possible outcomes in a CPP: the patient is diagnosed with the associated cancer, the patient is diagnosed with another type of cancer, or the patient is not diagnosed with cancer. In Norway, it is a national aim that at least 70% of all cancer patients are included in a CPP [13].

Although national criteria for inclusion in CPPs are stated clearly, the proportion of CPP patients who turn out not to have the associated cancer may still vary between hospital referral areas and subgroups of patients. High proportions of patients without the cancer diagnosis among the CPP patients may be an indication of open access or a wide funnel for inclusion into a CPP. Wide funnels into CPPs may also result in higher proportions of cancer patients included in CPPs and thus fewer cancer patients diagnosed and treated outside CPP.

The Norwegian healthcare system aims to provide equitable access to health care to all citizens, irrespective of their socioeconomic status (SES), place of residence, age, gender or ethnicity. Nevertheless, unwarranted geographic variation in health care utilisation has been documented by the Norwegian Healthcare Atlases [14] in a broad spectrum of services, in cancer treatment for the Norwegian elderly [15] and according to current official statistics [12]. Unwarranted variation is defined as differences in health care utilisation that cannot be explained by patient needs or preferences [16].

Nilssen et al. have previously studied cancer patients in CPPs in Norway [17]. However, the proportion of patients in CPPs without the cancer diagnosis, and socio-demographic factors associated with this proportion, are not known. Furthermore, the statistical relationship between the proportion of patients in a CPP without the cancer diagnosis and the proportion of cancer patients included in a CPP in Norway has not previously been studied.

The main aim of this study was to investigate the equality in access to CPPs in Norway. Based on individual data CPP patients without the cancer diagnosis and cancer patients included in CPP were studied. In addition, the relationships of patient factors such as place of residence, age, education, income, comorbidity and travel time to hospital were examined. A secondary aim was to investigate the relation between the proportion of CPP patients without the cancer diagnosis and the proportion of diagnosed cancer patients included in CPP across the geographic areas.

## Methods

### Study design and data sources

A national registry-based study was conducted linking data from the Norwegian Patient Registry (NPR), the Cancer Registry of Norway (CRN) and Statistics Norway (SSB). All Norwegian citizens have a unique 11-digit personal identifier that allows tracking of patients in time and between hospitals, regions and registries. Socioeconomic data from SSB were linked with the NPR and CRN data, by an encrypted serial number derived from the 11-digit personal identifier. The information from SSB consisted of yearly income and educational data. The NPR data consisted of patient characteristics (residential information, age and gender), hospital, diagnoses, and procedures. Cancer diagnosis and diagnosis date were obtained from the CRN data. All Norwegian hospitals, and all private specialists with public funding contracts, must submit data to NPR for registration and reimbursement purposes. All Norwegian cancer cases are to be reported to CRN.

The data included all Norwegian patients aged 18 years and above in CPPs for lung, colorectal, prostate or breast cancer (CPP patients) and patients diagnosed with lung, colorectal, prostate or breast cancer (cancer patients) in the period 1 January 2015 to 31 December 2017. Thus, two patient populations were analysed: i) CPP patients and the proportion without the relevant cancer and ii) cancer patients and the proportion included in the associated CPP.

### Definitions

Only the first cancer diagnosis (CRN data) and the first CPP (NPR data) for each patient were considered. The pathway type in the NPR data was matched on cancer diagnosis in the CRN data and vice versa. CPP patients without the associated cancer diagnoses, i.e., patients not diagnosed with cancer at all or patients diagnosed with another type of cancer, were defined as CPP patients without the cancer diagnosis.

Educational level was coded applying the international standard classification of education (ISCED) [18]. Larger numbers represented higher education levels; 0 represented less than primary education, and 8 indicated a doctorate or equivalent while 9 was not classified and regarded as missing. Education level in the analyses was recoded into three categories; low (0-2), medium (3-5) and high (6-8), where 3-5 is high school level.

After-tax income was calculated as total income minus assessed tax and negative transfers, with total income representing the sum of income as employee, income from self-employment, property income, capital income and transfers received. The after-tax income was index-adjusted to 2015 by the consumer price index (CPI) to account for inflation. From after-tax income a categorical income variable was defined with three categories; low (less than NOK 240 000), medium (NOK 240 000 - 400 000), high (more than NOK 400 000).

Comorbidity was measured by a modified version of the Charlson comorbidity index (CCI) [19], based on diagnostic codes (ICD-10) from hospitalisations within one year prior to the start of the CPP or the cancer diagnosis. The index was categorised into low (CCI=0), medium (CCI=1) and high (CCI >1).

The travel time by road was calculated from the patients’ municipality centre to the nearest hospital, and was categorised into short (less than 30 minutes), medium (30 to 60 minutes) and long (more than one hour).

The patients’ hospital referral areas were defined by place of residence and the corresponding geographic catchment areas served by the 21 Norwegian hospital trusts.

**Fig. 1:**
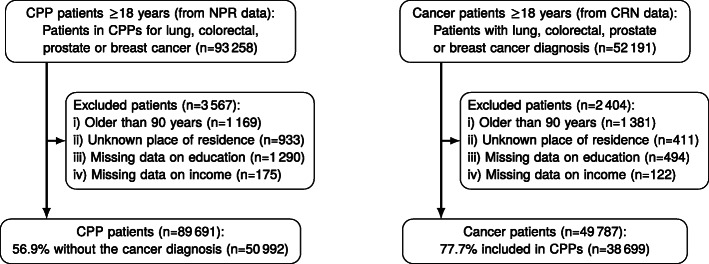
Flowchart for CPP patients and cancer patients, Norway 2015-2017

Patients with missing data on education or income, unknown place of residence or older than 90 years were excluded from the analysis (Fig. 1).

### Statistical analyses

Data were analysed using SAS 9.4 (SAS Institute, Cary NC).

The analyses for both CPP patients and cancer patients were stratified by gender and CPP type or cancer diagnosis. Separate analyses were conducted for i) patients in CPP and ii) patients with cancer diagnosis, for each of the six groups: lung (males and females), colorectal (males and females), prostate and breast (females only).

Two dichotomous outcomes were analysed by logistic regressions; i) among CPP patients: the proportion without the cancer diagnosis and ii) among cancer patients: the proportion included in the CPP. The following categorical independent variables were included in the statistical model: patients’ age in age intervals, level of income and education, comorbidity, travel time and hospital referral area. Age interval 60 to 69 years, high level of education and income, low comorbidity, short travel time and Akershus hospital referral area (the largest one in terms of number of patients) were set as reference categories. Wald tests were used to assess the significance of the independent variables and potential interactions. *P*-values for linear trends over categories were calculated for four independent variables; education, income, comorbidity and travel time, by separate analyses with all the other independent variables included as categorical without any assumptions of a linear trend. For main effects, *p*-values <0.05 were considered statistically significant. Because of the large number of potential interactions involved, interactions were considered statistically significant if *p*-value <0.01. However, no statistically significant interactions were observed.

The selection of the independent variables, i.e., adjusting for potential confounders and avoiding the Table II fallacy [20], was assessed in separate analyses by causal diagrams or the directed acyclic graph (DAG) methodology [21].

Age-adjusted proportions of CPP patients without the cancer diagnosis and of cancer patients included in CPP were calculated for the 21 hospital referral areas. The direct method of standardisation was applied. The reference populations were all CPP patients in the relevant CPP and all cancer patients with relevant cancer type, respectively, in the period. The Spearman’s rank correlation coefficient was computed between the age-adjusted proportion of CPP patients without the cancer diagnosis and the age-adjusted proportion of cancer patients included in CPP considering patients living in different hospital referral areas.

## Results

A total of 89 691 CPP patients and 49 787 cancer patients were eligible for analyses. Among all CPP patients considered, 56.9% were without the cancer diagnosis. This proportion was stable over the three years (Table 1). The proportion of CPP patients without the cancer diagnosis ranged from 43.9% for females in lung CPP to 69.3% for females in colorectal CPP. The proportion of CPP patients without the cancer diagnosis varied between the hospital referral areas (Table 1 and Table B1), from 45.4% to 69.4% for all four CPPs combined, and the variation in the proportions between the hospital referral areas was about two-fold across the CPP groups.

**Table 1:** Characteristics of CPP patients and cancer patients. Norway, 2015-2017

	Lung		Colorectal		Prostate	Breast	Total
	Male	Female	Male	Female			
	**CPP patients, n (% CPP patients without the cancer diagnosis)**						
Patients	7 461 (46.3%)	6 428 (43.9%)	15 802 (64.6%)	16 824 (69.3%)	19 897 (48.7%)	23 279 (56.5%)	89 691 (56.9%)
**Year**							
2015	2 375 (47.2%)	2 055 (43.5%)	4 726 (63.0%)	5 109 (68.1%)	6 113 (51.1%)	7 487 (56.8%)	27 865 (56.9%)
2016	2 521 (45.8%)	2 143 (44.1%)	5 660 (65.6%)	5 935 (69.6%)	6 992 (47.6%)	7 785 (56.9%)	31 036 (57.0%)
2017	2 565 (45.9%)	2 230 (44.1%)	5 416 (65.0%)	5 780 (70.1%)	6 792 (47.6%)	8 007 (56.0%)	30 790 (56.7%)
**Age group**							
Under 50	344 (78.5%)	294 (70.4%)	1 546 (83.4%)	1 772 (84.8%)	389 (70.2%)	8 511 (79.3%)	12 856 (80.1%)
50-59	838 (60.3%)	789 (50.6%)	2 480 (74.4%)	2 493 (78.2%)	2 841 (59.0%)	5 106 (52.5%)	14 547 (62.2%)
60-69	2 251 (45.3%)	1 989 (42.7%)	4 240 (62.9%)	4 088 (70.9%)	8 533 (51.3%)	4 888 (39.0%)	25 989 (52.8%)
70-79	2 778 (40.8%)	2 372 (39.0%)	4 816 (59.4%)	5 080 (65.7%)	6 720 (42.6%)	3 216 (40.9%)	24 982 (49.8%)
80-90	1 250 (42.2%)	984 (44.7%)	2 720 (56.9%)	3 391 (58.1%)	1 414 (35.1%)	1 558 (32.6%)	11 317 (48.5%)
**Income**							
Low	1 567 (42.0%)	2 936 (41.9%)	2 573 (63.1%)	6 933 (66.0%)	2 208 (47.0%)	6 402 (52.6%)	22 619 (55.2%)
Medium	4 259 (44.6%)	2 838 (44.3%)	8 258 (63.4%)	7 563 (70.5%)	9 696 (46.1%)	11 117 (56.9%)	43 731 (56.1%)
High	1 635 (54.9%)	654 (51.1%)	4 971 (67.5%)	2 328 (75.2%)	7 993 (52.2%)	5 760 (60.3%)	23 341 (59.9%)
**Education**							
Low	2 641 (42.5%)	2 553 (41.7%)	4 209 (65.2%)	5 312 (67.8%)	3 889 (45.9%)	5 132 (55.9%)	23 736 (55.6%)
Medium	3 683 (46.6%)	2 947 (43.1%)	7 759 (64.1%)	7 603 (68.3%)	10 108 (47.9%)	9 707 (53.1%)	41 807 (55.3%)
High	1 137 (54.4%)	928 (52.5%)	3 834 (65.0%)	3 909 (73.3%)	5 900 (51.8%)	8 440 (60.9%)	24 148 (60.7%)
**Comorbidity**							
Low	5 118 (48.5%)	4 725 (45.7%)	13 020 (64.8%)	14 701 (69.7%)	17 868 (48.8%)	21 991 (56.9%)	77 423 (57.5%)
Medium	1 484 (41.9%)	1 235 (37.7%)	1 739 (62.8%)	1 403 (69.1%)	1 420 (46.8%)	833 (51.9%)	8 114 (52.3%)
High	859 (41.1%)	468 (41.7%)	1 043 (65.4%)	720 (61.4%)	609 (49.6%)	455 (49.2%)	4 154 (52.9%)
**Travel time**							
Short	5 695 (47.0%)	5 052 (44.5%)	12 384 (64.9%)	13 417 (69.7%)	15 535 (49.4%)	19 024 (56.5%)	71 107 (57.3%)
Medium	1 018 (44.5%)	820 (40.1%)	2 052 (63.4%)	2 044 (66.4%)	2 646 (45.8%)	2 726 (58.6%)	11 306 (55.3%)
Long	747 (43.8%)	556 (43.7%)	1 366 (63.7%)	1 363 (69.8%)	1 714 (46.9%)	1 529 (53.6%)	7 275 (55.2%)
**Hospital referral area** ⋆							
Min *†*	32.4%	29.8%	42.5%	48.1%	28.9%	36.8%	45.4%
Max *†*	66.2%	61.3%	83.2%	84.0%	59.5%	73.1%	69.4%
SD	8.4%	7.7%	10.9%	10.0%	6.9%	9.2%	5.9%
EQ *‡*	2.0	2.1	2.0	1.7	2.1	2.0	1.5
	**Cancer patients, n (% cancer patients included in CPP)**						
Patients	5 076 (78.9%)	4 629 (77.9%)	6 836 (81.8%)	6 471 (79.8%)	15 864 (64.4%)	10 911 (92.7%)	49 787 (77.7%)
**Year**							
2015	1 650 (73.8%)	1 543 (73.0%)	2 252 (77.2%)	2 131 (75.5%)	5 136 (55.2%)	3 609 (89.3%)	16 321 (72.0%)
2016	1 682 (80.8%)	1 531 (78.8%)	2 307 (83.5%)	2 207 (81.8%)	5 378 (67.6%)	3 549 (94.5%)	16 654 (79.8%)
2017	1 744 (82.0%)	1 555 (82.0%)	2 277 (84.7%)	2 133 (82.1%)	5 350 (69.9%)	3 753 (94.3%)	16 812 (81.3%)
**Age group**							
Under 50	87 (85.1%)	105 (84.8%)	366 (69.9%)	382 (70.4%)	160 (71.3%)	1 886 (93.2%)	2 986 (85.7%)
50-59	396 (84.1%)	461 (84.4%)	752 (84.6%)	674 (80.7%)	1 667 (69.2%)	2 613 (92.9%)	6 563 (83.5%)
60-69	1 503 (81.5%)	1 376 (82.6%)	1 860 (84.4%)	1 434 (82.8%)	5 939 (69.7%)	3 207 (92.9%)	15 319 (79.9%)
70-79	2 039 (80.9%)	1 821 (79.5%)	2 350 (83.4%)	2 110 (82.5%)	5 920 (65.5%)	2 063 (92.2%)	16 303 (77.1%)
80-90	1 051 (69.0%)	866 (63.2%)	1 508 (77.7%)	1 871 (76.1%)	2 178 (42.4%)	1 142 (91.9%)	8 616 (67.8%)
**Income**							
Low	1 257 (72.2%)	2 273 (74.7%)	1 204 (78.7%)	2 984 (79.0%)	2 047 (56.8%)	3 336 (91.0%)	13 101 (77.2%)
Medium	2 942 (80.2%)	1 985 (80.1%)	3 702 (81.8%)	2 777 (80.3%)	8 199 (63.8%)	5 136 (93.3%)	24 741 (77.7%)
High	877 (84.4%)	371 (86.3%)	1 930 (83.8%)	710 (81.4%)	5 618 (67.9%)	2 439 (93.8%)	11 945 (78.4%)
**Education**							
Low	1 965 (77.3%)	1 978 (75.2%)	1 827 (80.1%)	2 181 (78.3%)	3 446 (61.0%)	2 473 (91.5%)	13 870 (76.0%)
Medium	2 474 (79.6%)	2 109 (79.6%)	3 393 (82.2%)	2 987 (80.7%)	8 000 (65.8%)	4 916 (92.7%)	23 879 (78.2%)
High	637 (81.5%)	542 (81.5%)	1 616 (82.9%)	1 303 (80.2%)	4 418 (64.4%)	3 522 (93.6%)	12 038 (78.8%)
**Comorbidity**							
Low	3 243 (81.4%)	3 179 (80.7%)	5 519 (83.1%)	5 498 (81.0%)	13 813 (66.2%)	10 219 (92.8%)	41 471 (79.3%)
Medium	1 110 (77.7%)	1 009 (76.3%)	820 (78.9%)	584 (74.1%)	1 352 (55.9%)	434 (92.4%)	5 309 (72.9%)
High	723 (70.0%)	441 (61.9%)	497 (72.6%)	389 (71.5%)	699 (43.9%)	258 (89.5%)	3 007 (65.0%)
**Travel time**							
Short	3 850 (78.4%)	3 620 (77.5%)	5 351 (81.2%)	5 129 (79.3%)	12 291 (64.0%)	8 915 (92.8%)	39 156 (77.6%)
Medium	708 (79.9%)	603 (81.3%)	900 (83.6%)	842 (81.5%)	2 202 (65.1%)	1 217 (92.8%)	6 472 (78.1%)
Long	518 (81.3%)	406 (77.1%)	585 (84.8%)	500 (82.2%)	1 371 (66.2%)	779 (91.1%)	4 159 (78.4%)
**Hospital referral area** ⋆							
Min *†*	69.0%	67.6%	69.4%	67.2%	44.8%	82.6%	72.7%
Max *†*	87.7%	86.8%	89.9%	88.1%	74.6%	98.4%	84.5%
SD	5.5%	5.9%	5.1%	4.8%	8.5%	3.6%	3.8%
EQ *‡*	1.3	1.3	1.3	1.3	1.7	1.2	1.2

*†* Min and Max are minimum and maximum proportions in the 21 hospital referral areas. *‡* EQ - Extremal quotient, EQ=Max/Min. ⋆ For details on the 21 hospital referral areas see Appendix B (Table B1)

In total 77.7% of the cancer patients were included in relevant CPPs, and the proportion increased from 72.0% in 2015 to 81.3% in 2017 (Table 1). The proportion of cancer patients included in CPP varied from 64.4% for prostate cancer to 92.7% for breast cancer, and between the hospital referral areas from 72.7% to 84.5% for all four types of cancer combined (Table 1 and Table B1) and more markedly across the cancer groups.

### Relationships with age, income, education, comorbidity and hospital referral area

The main results from the multivariate logistic regression analysis are the effects after mutually adjusting for all the other independent variables, and adjustment for the independent variables according to the DAG methodology showed largely similar results.

#### Age group

The adjusted analyses for all CPP patients showed an inverse age gradient, indicating lower odds ratio of not receiving the cancer diagnosis with increasing age (Table 2). The only exceptions were the oldest patients (80-90 years) in CPP for lung cancer. An inverse age gradient was not consistently found in the adjusted analyses for the cancer patients (Table 2). However, the oldest patients (80-90 years) had lower odds ratio of being included in CPP for all cancer diagnoses except for breast cancer, along with the youngest colorectal cancer patients (under 50).

**Table 2:** Associations between age, income, education, comorbidity, travel time and hospital referral area and the odds ratios of not receiving the cancer diagnosis in CPP patients and the odds ratio of being included in CPP in cancer patients. Norway, 2015-2017. Analyses with mutual adjustment for all variables included

	Lung		Colorectal		Prostate	Breast
	Male	Female	Male	Female		
	**CPP patients: odds ratio of not receiving the cancer diagnosis.** OR (95% CI)					
**Age group**						
Under 50	4.19 (3.19-5.52)	2.89 (2.20-3.80)	2.91 (2.50-3.38)	2.29 (1.97-2.67)	2.18 (1.75-2.73)	5.97 (5.51-6.47)
50-59	1.79 (1.52-2.12)	1.31 (1.11-1.55)	1.67 (1.49-1.87)	1.47 (1.30-1.65)	1.36 (1.25-1.49)	1.76 (1.62-1.90)
60-69	1.00 (ref)	1.00 (ref)	1.00 (ref)	1.00 (ref)	1.00 (ref)	1.00 (ref)
70-79	0.88 (0.78-0.99)	0.87 (0.77-0.99)	0.86 (0.79-0.94)	0.81 (0.73-0.88)	0.71 (0.66-0.75)	1.03 (0.94-1.13)
80-90	0.97 (0.84-1.12)	1.13 (0.96-1.33)	0.78 (0.70-0.87)	0.59 (0.53-0.65)	0.50 (0.45-0.57)	0.69 (0.61-0.78)
*p*-value	<0.001	<0.001	<0.001	<0.001	<0.001	<0.001
**Income**						
Low	0.76 (0.65-0.90)	0.94 (0.77-1.14)	0.96 (0.85-1.07)	0.96 (0.85-1.09)	1.02 (0.92-1.13)	1.15 (1.05-1.26)
Medium	0.82 (0.72-0.93)	0.91 (0.76-1.10)	0.99 (0.91-1.08)	0.99 (0.89-1.12)	0.96 (0.90-1.03)	1.10 (1.02-1.18)
High	1.00 (ref)	1.00 (ref)	1.00 (ref)	1.00 (ref)	1.00 (ref)	1.00 (ref)
*p*-value^†^	0.001	0.803	0.493	0.444	0.828	0.003
**Education**						
Low	0.69 (0.60-0.81)	0.74 (0.62-0.88)	1.03 (0.93-1.14)	1.00 (0.89-1.11)	0.85 (0.78-0.93)	1.08 (0.99-1.17)
Medium	0.80 (0.69-0.92)	0.79 (0.67-0.92)	1.00 (0.92-1.09)	0.97 (0.88-1.07)	0.90 (0.84-0.96)	0.95 (0.89-1.02)
High	1.00 (ref)	1.00 (ref)	1.00 (ref)	1.00 (ref)	1.00 (ref)	1.00 (ref)
*p*-value^†^	<0.001	0.003	0.54	0.955	<0.001	0.146
**Comorbidity**						
Low	1.00 (ref)	1.00 (ref)	1.00 (ref)	1.00 (ref)	1.00 (ref)	1.00 (ref)
Medium	0.90 (0.80-1.02)	0.79 (0.69-0.91)	1.10 (0.99-1.23)	1.14 (1.01-1.29)	1.05 (0.94-1.18)	1.29 (1.11-1.49)
High	0.90 (0.77-1.05)	0.95 (0.78-1.15)	1.20 (1.05-1.38)	0.92 (0.78-1.08)	1.25 (1.06-1.48)	1.35 (1.11-1.65)
*p*-value^†^	0.07	0.025	0.003	0.826	0.008	<0.001
**Travel time**						
Short	1.00 (ref)	1.00 (ref)	1.00 (ref)	1.00 (ref)	1.00 (ref)	1.00 (ref)
Medium	1.03 (0.89-1.19)	0.92 (0.78-1.08)	1.01 (0.90-1.12)	0.93 (0.83-1.04)	0.94 (0.86-1.03)	1.13 (1.03-1.24)
Long	0.97 (0.80-1.16)	0.94 (0.76-1.16)	0.98 (0.86-1.12)	1.02 (0.89-1.18)	0.92 (0.82-1.04)	1.08 (0.95-1.23)
*p*-value^†^	0.876	0.353	0.825	0.718	0.09	0.026
**Hospital referral area**^*‡*^						
*p*-value	<0.001	<0.001	<0.001	<0.001	<0.001	<0.001
	**Cancer patients: odds ratio of being included in CPP.** OR (95% CI)					
**Age group**						
Under 50	1.14 (0.61-2.10)	1.05 (0.60-1.85)	0.41 (0.32-0.54)	0.48 (0.37-0.63)	0.98 (0.69-1.39)	0.98 (0.78-1.24)
50-59	1.14 (0.84-1.55)	1.07 (0.80-1.43)	0.98 (0.77-1.24)	0.83 (0.65-1.05)	0.96 (0.85-1.08)	0.98 (0.80-1.20)
60-69	1.00 (ref)	1.00 (ref)	1.00 (ref)	1.00 (ref)	1.00 (ref)	1.00 (ref)
70-79	1.02 (0.85-1.21)	0.87 (0.73-1.05)	0.99 (0.83-1.17)	1.06 (0.89-1.28)	0.84 (0.78-0.92)	1.00 (0.81-1.25)
80-90	0.55 (0.46-0.67)	0.40 (0.33-0.49)	0.73 (0.61-0.88)	0.74 (0.62-0.89)	0.34 (0.31-0.38)	1.04 (0.80-1.35)
*p*-value	<.001	<.001	<.001	<.001	<.001	0.996
**Income**						
Low	0.59 (0.46-0.75)	0.64 (0.45-0.90)	0.77 (0.62-0.96)	0.85 (0.67-1.09)	0.82 (0.72-0.92)	0.77 (0.60-0.98)
Medium	0.91 (0.73-1.14)	0.73 (0.53-1.02)	0.87 (0.73-1.02)	0.88 (0.71-1.11)	0.98 (0.90-1.06)	0.99 (0.80-1.22)
High	1.00 (ref)	1.00 (ref)	1.00 (ref)	1.00 (ref)	1.00 (ref)	1.00 (ref)
*p*-value^†^	<0.001	0.009	0.017	0.257	0.005	0.014
**Education**						
Low	0.93 (0.73-1.19)	0.97 (0.74-1.28)	0.87 (0.71-1.05)	0.91 (0.75-1.11)	1.03 (0.93-1.15)	0.84 (0.67-1.05)
Medium	0.92 (0.73-1.16)	1.04 (0.80-1.36)	0.94 (0.80-1.11)	0.99 (0.83-1.19)	1.12 (1.03-1.22)	0.95 (0.78-1.14)
High	1.00 (ref)	1.00 (ref)	1.00 (ref)	1.00 (ref)	1.00 (ref)	1.00 (ref)
*p*-value^†^	0.699	0.555	0.142	0.281	0.407	0.124
**Comorbidity**						
Low	1.00 (ref)	1.00 (ref)	1.00 (ref)	1.00 (ref)	1.00 (ref)	1.00 (ref)
Medium	0.82 (0.69-0.98)	0.80 (0.67-0.96)	0.76 (0.63-0.91)	0.68 (0.55-0.83)	0.73 (0.65-0.82)	0.96 (0.67-1.40)
Much	0.58 (0.48-0.70)	0.41 (0.33-0.51)	0.56 (0.45-0.70)	0.61 (0.48-0.77)	0.50 (0.43-0.59)	0.72 (0.47-1.10)
*p*-value^†^	<0.001	<0.001	<0.001	<0.001	<0.001	0.165
**Travel time**						
Short	1.00 (ref)	1.00 (ref)	1.00 (ref)	1.00 (ref)	1.00 (ref)	1.00 (ref)
Medium	1.09 (0.88-1.35)	1.19 (0.94-1.51)	0.96 (0.78-1.18)	0.98 (0.80-1.19)	1.06 (0.96-1.18)	1.11 (0.87-1.42)
Long	1.05 (0.80-1.37)	0.85 (0.64-1.13)	1.10 (0.85-1.43)	0.98 (0.75-1.28)	1.21 (1.06-1.39)	0.78 (0.59-1.04)
*p*-value^†^	0.535	0.774	0.652	0.815	0.006	0.295
**Hospital referral area**^*‡*^						
*p*-value	<0.001	<0.001	<0.001	<0.001	<0.001	<0.001

*†P*-value for linear trend. *‡* OR estimates with 95% CI for the hospital referral areas are shown in Appendix B (Table B2)

#### Income

A positive income gradient was found for male lung CPP patients, indicating increased odds ratio of not receiving the cancer diagnosis with increasing income. However, for breast CPP patients the opposite was true. For the cancer patients, a positive income gradient was found, indicating increased odds ratio of being included in CPP with increasing income, although the relationship was not statistically significant for female colorectal cancer patients.

#### Education

A positive education gradient was found for patients for lung CPP (male and female) and prostate CPP, indicating increased odds ratio of not receiving the cancer diagnosis with increasing level of education. For cancer patients no education gradient was found.

#### Comorbidity

A positive comorbidity gradient was found for patients in colorectal CPP (male), prostate CPP and breast CPP, suggesting increased odds ratio of not receiving the cancer diagnosis with increasing comorbidity. For cancer patients a clear negative comorbidity gradient was found for all cancer groups, except breast cancer patients, i.e., decreased odds ratio of being included in CPP with increasing comorbidity.

#### Travel time

No gradient for travel time was found for CPP patients. For cancer patients a positive gradient for travel time was found for patients with prostate cancer, the longer travel time to hospital, the higher the odds ratio of being included in CPP.

#### Hospital referral area

Substantial differences were found between the hospital referral areas both regarding the odds ratio of not receiving the cancer diagnosis among all the CPP patients and in the odds ratio of being included in a CPP among cancer patients (Table 2 and Table B2).

### Relationships between the proportions

**Fig. 2:**
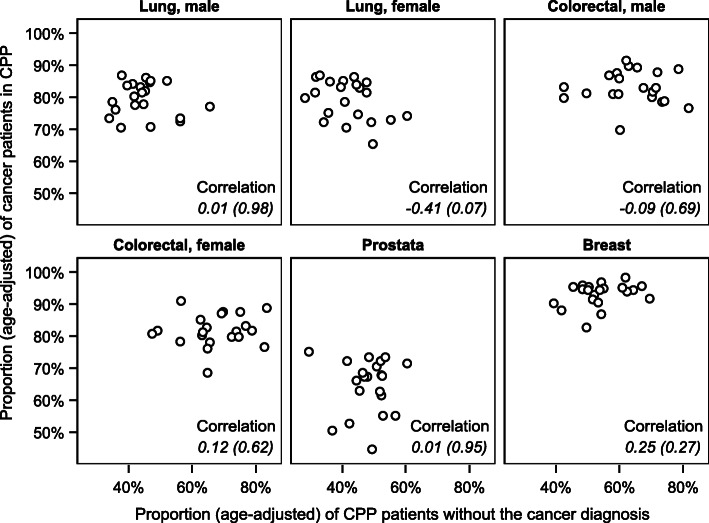
Scatter plots for age-adjusted proportions of patients in CPP without the cancer diagnosis and age-adjusted proportions of cancer patients included in CPP in the 21 hospital referral areas. Spearman’s correlation coefficients (*p*-value)

Correlation analysis comparing the age-adjusted proportions of CPP patients without the cancer diagnosis with the age-adjusted proportions of cancer patients included in CPP across the hospital referral areas did not show definite positive relations for any cancer site (Fig. 2).

**Fig. 3:**
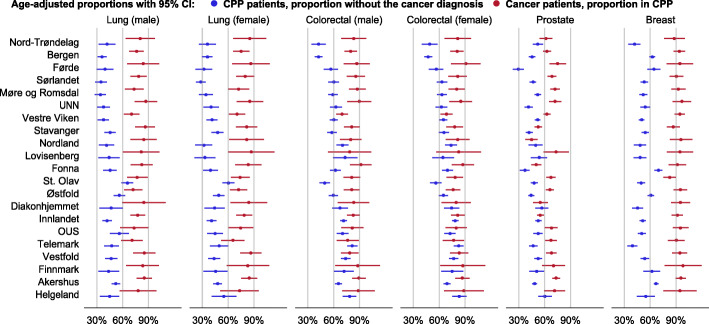
Patients in CPP without the cancer diagnosis (blue) and cancer patients in CPP (red), age-adjusted proportions with 95% confidence intervals in the 21 hospital referral areas, ordered by the rank of the total proportion for CPP patients

Correlation analysis comparing the age-adjusted proportions of CPP patients without the cancer diagnosis in the 21 hospital referral areas across the six CPP groups showed some positive associations. Associations were found between the proportions for prostate CPP and colorectal (both male and female) CPP (Table B3). Also, comparing the age-adjusted proportion of cancer patients included in CPP in the 21 hospital referral areas across the six cancer groups showed some positive associations. Associations were found between the proportions for female colorectal cancer and lung (both male and female) cancer (Table B3). However, most correlation coefficients, except for those correlating proportions in men and women for the same cancer type, were relatively low. The overall impression is that neither the proportions of CPP patients without the cancer diagnosis nor the proportions of cancer patients included in CPP across the cancer groups are consistently ranked in the hospital referral areas (Fig. 3).

## Discussion

This study, which includes complete CPP and cancer data for three years in Norway, showed that the patient’s age and place of residence were the two most important factors for describing variation in proportions of CPP patients without the cancer diagnosis and proportions of cancer patients included in CPPs. The proportions varied substantially across the different types of CPPs and across the different cancer groups. As the probability of not receiving the cancer diagnosis for a patient in a CPP is related to both the risk of cancer and the probabilities of being included in a CPP for those with and without the cancer (See Appendix A. Conditional probabilities), the interpretations must account for these factors.

### CPP guidelines

In general, the CPP guidelines ensure that all patients should have equal access to CPPs in Norway. The highest proportions of CPP patients without the cancer diagnosis were found in CPPs for colorectal cancer (65% and 69% for male and female, respectively) and breast cancer (57%). Less than 50% of the patients in CPPs for lung and prostate ended up without the cancer diagnosis. These differences may be explained by the “filter functions” for CPP for prostate and lung cancer. Only specialists in urology include patients to CPP for prostate cancer and the guidelines specify that patients should not be referred to CPP for prostate cancer when treatment is not appropriate due to age or comorbidity. Before inclusion in CPP for lung cancer, an X-ray image of the lungs is required in addition to respiratory symptoms. Similar filter functions are lacking for CPP for colorectal and breast cancer. The lowest proportion of cancer patients included in CPP was found for prostate cancer patients (64%).

### Age and comorbidity

The highest proportion of CPP patients without the cancer diagnosis was found for the youngest patients and the proportion decreased with increasing age. The negative age gradient found in CPP patients without the cancer diagnosis might be explained by an increasing cancer incidence by age with lower probability for younger patients to have a cancer diagnosis. Given the same symptoms, an older patient is more likely to have cancer compared to a younger patient. A high proportion of CPP patients without the cancer diagnosis among the younger suggests that inclusion in CPPs in Norway follows the guidelines and is based on symptoms, signs and tests rather than the expected chance of detecting cancer.

Among cancer patients, except for breast cancer, the oldest patients had lower odds ratio of being included in a CPP than patients in the reference category, patients aged 60-69. This is in line with studies from Norway and Denmark [17, 22, 23]. One explanation might be that the elderly have higher comorbidity and more contact with the health care system. Cancer may be diagnosed during follow-up of other illnesses. The youngest colorectal cancer patients (under 50 years) also had lower odds ratio of being included in CPP, probably because the symptoms of younger colorectal cancer patients are less specific and harder to identify. Interestingly, more than 90% of the breast cancer patients were included in CPP, and no age differences were found. This high proportion and equality among the age groups are most likely due to the uniform structure and practice of breast diagnostic centres in Norway and the national breast screening programme for women aged 50 to 69. In all cancer patients, the probability of being included in CPP decreased with higher level of comorbidity, consistent with the findings of Nilssen et al. [17]. According to guidelines, patients with prostate cancer and high comorbidity or high age are not to be included in CPP if curative treatment is not relevant. Other patient groups may be considered similarly.

### Income and education

The effects of income and education were not consistent across the CPP groups. In a report on social inequalities and cancer in Norway [24], associations between both income and education level and the risk of cancer were found. The risk of cancer increased with low level of income for males for lung and colorectal cancer, and increased risk of cancer with low level of education was found for lung cancer and among females for colorectal cancer. In contrast, increased risk of cancer with high levels of income and education was found for prostate and breast cancer. The observed differences in the odds ratio of not receiving the cancer diagnosis between the income groups for CPPs for lung (male) and breast cancer, in this study, might be due to different risks of cancer in the income groups between the CPPs.

The probability of being referred to a CPP, both for those with and without cancer, may also differ between income and education groups. Based on data in this study, only the probability for those with cancer can be estimated (Appendix A). The observed differences in income and education gradients for CPP patients without the cancer diagnosis may diminish when the risk of cancer and the probabilities of being included in a CPP are taken into consideration. A systematic review concluded that women in Europe from more socioeconomically deprived areas were less likely to attend breast cancer screening [25]. Higher income and education groups might be more eager to be included in CPPs in order to assess their cancer symptoms. It is documented that patients from higher social classes communicate more actively with clinicians [26]. Therefore, based on the present data, it is not possible to state consistent differences in the proportions of CPP patients without the cancer diagnosis in different levels of income and education.

For the cancer patients, consistent differences were found. The proportion of cancer patients included in CPPs was highest in the patients with high income, which is in line with a recent Norwegian study [17]. Other studies have shown that patients’ socioeconomic status is associated with an increased likelihood of access to cancer care [27, 28]. However, in accordance with results of Nilssen et al. [17], we found no such effect of education for any of the cancer groups.

### Place of residence

Substantial variation between the hospital referral areas was found, both for the proportion of CPP patients without the cancer diagnosis and the proportion of cancer patients included in CPP. The proportions in neighbouring hospital referral areas were not more similar than for more distant hospital referral areas. The observed variation between the hospital referral areas is probably related to both differences in clinical practice and differences in capacity. Unwarranted variation in health care is mainly due to services that can be defined as preference-sensitive or supply-sensitive [29]. Preference-sensitive care represents clinical practice, references and beliefs of a single clinician or department rather than a clear evidence-based approach. Supply-sensitive care refers to local capacity of health care resources, such as out-patients clinics and diagnostic work-ups. In addition, the hospital trusts have public funding contracts with private specialists in order to secure sufficient capacity, and different utilisation of private specialists among the health care trusts may contribute to the observed variation. Additionally, hospitals may have included patients in CPPs at somewhat different points in the diagnostic and staging process, although clear guidelines exist. This may also affect the observed variation.

One might expect that a hospital referral area with a high proportion of patients without the cancer diagnosis in CPPs, also has a high proportion of diagnosed cancer patients included in CPPs. However, such associations were not found, suggesting that a wide funnel into CPPs does not necessarily lead to higher proportions of cancer patients included in CPPs. This finding is in line with a study from England on variation in referral threshold and variation in the accurate selection of patients in fast-track referrals from GPs for possible cancer [30]. It was found that widening the funnel did not increase the proportion of cancer patients included in CPP. However, it did result in a large increase in demand for diagnostic services with a possible risk of over-diagnosis and over-treatment. Therefore, tools that focus on increasing diagnostic accuracy are probably more effective than applying wider funnels to increase the proportion of cancer patients included in CPPs. A Nordic initiative for prostate cancer can serve as an example: application of a new blood-based test in addition to PSA resulted in promising results for a more accurate selection of patients into CPP for prostate cancer [31–33].

It was not consistently the same hospital referral areas that had the highest or lowest proportions of CPP patients without the cancer diagnosis or the highest or lowest proportions of cancer patients included in CPP. As the diagnostic work-ups for the different CPPs are done at different departments/units in the hospitals, it is reasonable to see varying degrees of inclusion to CPP in the hospital referral areas across the different CPPs. A hospital might have lower threshold for including patients in CPP in some areas due to capacity or clinical practice (for example, the gastroenterological and lung clinics) while having higher threshold for other areas due to capacity constraints or stricter clinical practice (for example, prostate cancer).

### Experiences with CPPs

CPPs in Norway were introduced as a joint action from health politicians, health bureaucrats and clinicians as an initiative from the Ministry of Health and patient organisations. The experiences with CPPs in Norway are so far positive [34, 35]. In Norway, decreased waiting times have been observed before and during implementation of CPPs [36]. Studies from Denmark [3, 37] and Sweden [38] have documented reduced waiting times, and a study from Denmark showed increased survival among patients in CPP compared to patients not in CPP for some cancer sites [23]. It is worth noting that Denmark started CPP eight years before Norway, a period in which cancer care made great progress in Norway.

CPPs may also have some unintended consequences. Wilkens et al. discuss challenges with possible crowding-out effects as a result of implementing CPP [5]. A Norwegian study, with interviews of physicians and patients, indicated possible crowding-out effects as a result of the standardised target times in CPPs [35, 39]. In a qualitative study on CPP in Sweden, implementation of CPPs was accompanied by unintended effects such as longer waiting times for other patients and patient groups in need of the same health care resources [40]. However, in Norway these types of crowding-out effects are not observed in quantitative data, such as national data for waiting times [41].

### Strengths and limitations

The strength of this study is the use of individualised data for the complete Norwegian population of CPP patients and cancer patients for three years. The completeness of data eliminates selection bias. However, there are some limitations.

Firstly, the proportions studied empirically in this paper reflect two underlying conditional probabilities, the probability of not having cancer given that an individual is assigned to a CPP, and the probability of being included in a CPP given that the patient is diagnosed with cancer. The probability of not having cancer for an individual included in a CPP can be expressed in terms of the overall probability of cancer and the ratio of the probabilities of being assigned to a CPP for those with and without cancer (Appendix A). However, it is not possible to estimate all relevant terms involved with the data available in the current study. Such analysis requires data on the entire Norwegian population, including those not in contact with the hospitals. It is important to note that the relationships presented in Appendix A and discussed above do not invalidate the findings as presented; the relationships reflect the associations as they are in Norwegian cancer care. The explanations for the findings are not, however, self-evident and may be open for discussion.

Secondly, it was not possible in this study to evaluate at which point in the diagnostic and staging work-up patients are included in CPPs. The possibility that cancer patients have been retrospectively included in CPPs cannot be ruled out. However, this probably contributes only to a small degree to the observed geographic variation.

Thirdly, the presented results are mutually adjusted for all the predictors, i.e., there is a risk of Table II fallacy. However, adjusting the predictors according to a DAG framework basically reproduced the results in Table 2.

## Conclusion

Among the CPP patients, the youngest patients had the highest probability of not receiving the cancer diagnosis, and among the cancer patients the oldest patients had the lowest probability of being included in a CPP. This is most likely due to increasing risk of cancer and increasing contacts with the health care system with age. The lack of consistent differences among CPP patients according to income, education, comorbidity and travel time to hospital with regard to the proportion not diagnosed with cancer is encouraging and suggests that CPP as a health service in Norway is equitably distributed. Increasing diagnostic accuracy is probably a more effective way to increase the proportions of cancer patients included in CPPs, as lowering the threshold for referring patients into CPPs did not have an effect. Lowering the threshold may also lead to an increase in demand for diagnostic services with a possible risk of over-diagnosis, over-treatment and crowding-out effects for other patients. Unwarranted variation according to patients’ place of residence was documented, both for CPP patients without the cancer diagnosis and for cancer patients included in CPP. This variation is probably related to both differences in clinical practice and capacity at the Norwegian hospitals and should be further investigated.

## Supplementary Information

**Additional file 1** Appendix

## Data Availability

The original data were not collected by the authors but made available by record-linkage, using the unique 11 digits personal ID, between the Norwegian Patient Registry (NPR), Cancer Registry of Norway (CRN) and the Statistics Norway (SSB) (NPR ref. 18/28584-13, CRN ref. 18/265 DU-3294 and SSB ref. W19/0477). Individual-level health data are, by definition, considered to be sensitive information in the Norwegian legislation, even if de-identified and strict confidentiality requirements prevent sharing of data in public repositories. According to a contract signed with the NPR, CRN and the SSB, the project is not allowed to forward data, or subsets of data, to other researchers, except project members named in the Data Protection Impact Assessment (DPIA). Furthermore, we are required to delete the linked data set by 31 December 2023. However, any researcher with approval of an exemption from professional secrecy requirements for the use of personal health data in research from the Regional Committee for Medical and Health Research Ethics (REK) would be able to create an almost identical (updated) dataset by applying to the NPR, the CRN and the SSB.

## Abbreviations

CCI: Charlson comorbidity index
CPP: Cancer patient pathways
CRN: Cancer Registry of Norway
DAG: Directed acyclic graph GP: General practitioner
ICD-10: International Statistical Classification of Diseases and Related Health Problems diagnosis codes
ISCED: International standard classification of education
NPR: Norwegian Patient Registry
SES: Socioeconomic status
SSB: Statistics Norway
