# Supplementary material for: Equitable access to cancer patient pathways in Norway – a national registry-based study
Source: BMC Health Serv Res. 2021 Nov 25;21:1272. doi: 10.1186/s12913-021-07250-1 (PMC8613926; doi:10.1186/s12913-021-07250-1)
Supplement: Supplementary file 1 — Additional file 1 Appendix [file 12913_2021_7250_MOESM1_ESM.pdf]

Appendix

A. Conditional probabilities

Consider what happens during the study period to an individual selected at random from a subgroup in the population. Let “Cancer” represent the event that the individual actually has cancer of a particular type, and let “CPP” represent the event that the individual is referred to the corresponding cancer patient pathway.

The probability of not having this type of cancer, conditional on the individual being referred to the corresponding CPP, can be expressed as

$$P(\overline{\text{Cancer}}|\text{CPP}) = \frac{P(\text{CPP and }\overline{\text{Cancer}})}{P(\text{CPP})}$$

Applying Bayes’ theorem

$$P(\overline{\text{Cancer}}|\text{CPP}) = \frac{P(\text{CPP}|\overline{\text{Cancer}})P(\overline{\text{Cancer}})}{P(\text{CPP}|\text{Cancer})P(\text{Cancer}) + P(\text{CPP}|\overline{\text{Cancer}})P(\overline{\text{Cancer}})}$$

Dividing both numerator and denominator with $P(\text{CPP}|\overline{\text{Cancer}})$

$$P(\overline{\text{Cancer}}|\text{CPP}) = \frac{P(\overline{\text{Cancer}})}{\frac{P(\text{CPP}|\text{Cancer})}{P(\text{CPP}|\overline{\text{Cancer}})}P(\text{Cancer}) + P(\overline{\text{Cancer}})}$$

Finally, dividing both numerator and denominator with $P(\overline{\text{Cancer}})$, the probability of not having this type of cancer, conditional on the individual being referred to the corresponding CPP, can be written as

$$P(\overline{\text{Cancer}}|\text{CPP}) = \frac{1}{\frac{P(\text{CPP}|\text{Cancer})}{P(\text{CPP}|\overline{\text{Cancer}})}\frac{P(\text{Cancer})}{P(\overline{\text{Cancer}})} + 1}$$

This equation expresses the first conditional probability of interest, the probability of not having cancer conditional on the individual being referred to the corresponding CPP; $P(\overline{\text{Cancer}}|\text{CPP})$, in terms of the second one, the probability of being referred to CPP conditional on the individual having the corresponding cancer; $P(\text{CPP}|\text{Cancer})$. But the expression also involves the overall probability of having the cancer in question; $P(\text{Cancer})$, and the conditional probability of being referred to the CPP for someone without the cancer; $P(\text{CPP}|\overline{\text{Cancer}})$.

This study is based on data on all Norwegian patients in CPPs and all Norwegian patients with a cancer diagnosis. It is not possible to make statements about associations with the overall probability of having cancer or the probability of being referred to a

CPP for a person without cancer. That would require data on the entire Norwegian population. Thus, despite the mathematical relationship described above, it is still relevant to examine associations with both proportions by a statistical approach, as carried out in this work.

A wide funnel into a CPP will in general correspond to large values of both probabilities $P(\text{CPP}|\text{Cancer})$ and $P(\text{CPP}|\overline{\text{Cancer}})$. The expression above shows that the ratio between these probabilities is essential. In fact, the probability of not having cancer for someone referred to a CPP is a decreasing function of this ratio. Studying associations between the proportion of CPP patients without cancer and other factors is for this reason largely the same as comparing the width of the funnel into CPP for cancer and non-cancer patients. However, the proportion is also a decreasing function of the basic probability of cancer, so results may in addition reflect associations with cancer incidence rates.

B. Supplementary tables and figures

Table B1: CPP patients and cancer patients, by hospital referral area. Norway, 2015-2017.

	Lung		Colorectal		Prostate	Breast	Total
	Male	Female	Male	Female			
**CPP patients, n (% CPP patients diagnosed without cancer)**							
Number of patients	7 461 (46.3%)	6 428 (43.9%)	15 802 (64.6%)	16 824 (69.3%)	19 897 (48.7%)	23 279 (56.5%)	89 691 (56.9%)
**Hospital referral area**							
Finnmark	158 (43.7%)	135 (46.7%)	264 (73.5%)	254 (78.0%)	291 (51.2%)	307 (58.6%)	1 409 (60.5%)
UNN	306 (37.3%)	247 (38.9%)	573 (62.8%)	565 (63.0%)	741 (41.8%)	826 (50.8%)	3 258 (50.8%)
Nordland	247 (42.1%)	160 (31.3%)	623 (70.0%)	638 (74.6%)	315 (48.9%)	475 (45.7%)	2 458 (58.5%)
Helgeland	136 (54.4%)	132 (54.5%)	536 (79.7%)	555 (83.4%)	422 (59.5%)	291 (51.9%)	2 072 (69.4%)
Nord-Trøndelag	196 (44.9%)	169 (36.7%)	293 (43.0%)	270 (51.1%)	621 (51.0%)	423 (38.8%)	1 972 (45.4%)
St. Olav	568 (66.2%)	517 (61.3%)	610 (48.9%)	614 (54.4%)	1 200 (48.8%)	993 (47.8%)	4 502 (53.0%)
Møre-Romsdal	322 (35.7%)	238 (33.6%)	859 (57.6%)	851 (61.8%)	1 460 (53.0%)	1 082 (49.8%)	4 812 (52.6%)
Førde	182 (40.1%)	134 (37.3%)	358 (56.4%)	343 (58.0%)	367 (28.9%)	527 (61.7%)	1 911 (50.0%)
Bergen	533 (35.6%)	418 (35.2%)	866 (42.5%)	904 (48.1%)	1 359 (45.3%)	2 257 (63.9%)	6 337 (50.4%)
Fonna	341 (46.0%)	230 (41.7%)	674 (61.9%)	724 (69.3%)	495 (36.4%)	1 342 (73.1%)	3 806 (61.3%)
Stavanger	486 (46.1%)	430 (47.9%)	804 (59.0%)	810 (64.9%)	1 024 (43.1%)	1 239 (55.7%)	4 793 (53.4%)
Østfold	539 (56.4%)	450 (49.1%)	876 (59.0%)	943 (64.1%)	1 447 (43.8%)	1 659 (61.9%)	5 914 (55.9%)
Akershus	805 (53.2%)	722 (48.8%)	1 578 (66.3%)	1 666 (70.4%)	2 079 (48.5%)	3 493 (69.8%)	10 343 (62.3%)
OUS	226 (55.8%)	238 (44.1%)	657 (70.6%)	723 (73.4%)	725 (52.7%)	973 (49.7%)	3 542 (59.1%)
Lovisenberg	141 (48.2%)	95 (42.1%)	224 (72.3%)	213 (67.6%)	269 (54.3%)	396 (52.5%)	1 338 (57.4%)
Diakonhjemmet	121 (51.2%)	142 (46.5%)	370 (68.4%)	438 (73.1%)	432 (56.7%)	554 (43.7%)	2 057 (57.8%)
Innlandet	582 (42.1%)	535 (41.5%)	1 842 (71.9%)	2 155 (79.4%)	1 592 (53.8%)	1 646 (50.2%)	8 352 (62.1%)
Vestre Viken	451 (37.0%)	455 (40.4%)	1 117 (60.6%)	1 166 (64.9%)	1 895 (51.1%)	1 989 (49.3%)	7 073 (52.8%)
Vestfold	402 (48.5%)	385 (44.4%)	1 005 (74.2%)	1 140 (76.5%)	1 050 (52.2%)	1 053 (51.9%)	5 035 (61.1%)
Telemark	278 (47.8%)	234 (48.3%)	985 (83.2%)	1 091 (84.0%)	799 (48.4%)	590 (36.8%)	3 977 (65.0%)
Sørlandet	441 (32.4%)	362 (29.8%)	688 (58.6%)	761 (62.7%)	1 314 (47.7%)	1 164 (52.1%)	4 730 (50.0%)
**Cancer patients, n (% cancer patients included in CPP)**							
Number of patients	5 076 (78.9%)	4 629 (77.9%)	6 836 (81.8%)	6 471 (79.8%)	15 864 (64.4%)	10 911 (92.7%)	49 787 (77.7%)
**Hospital referral area**							
Finnmark	104 (85.6%)	89 (80.9%)	83 (84.3%)	64 (87.5%)	196 (71.9%)	129 (98.4%)	665 (83.5%)
UNN	219 (87.7%)	174 (86.8%)	245 (86.9%)	251 (83.3%)	597 (72.2%)	420 (96.7%)	1 906 (84.1%)
Nordland	174 (82.2%)	137 (80.3%)	233 (80.3%)	198 (81.8%)	359 (44.8%)	269 (95.9%)	1 370 (74.5%)
Helgeland	80 (77.5%)	88 (68.2%)	126 (86.5%)	110 (83.6%)	243 (70.4%)	149 (94.0%)	796 (79.6%)
Nord-Trøndelag	135 (80.0%)	129 (82.9%)	203 (82.3%)	162 (81.5%)	495 (61.4%)	290 (89.3%)	1 414 (76.2%)
St. Olav	252 (76.2%)	269 (74.3%)	386 (80.8%)	355 (78.9%)	918 (67.0%)	626 (82.6%)	2 806 (75.4%)
Møre-Romsdal	280 (73.9%)	214 (73.8%)	415 (87.7%)	407 (79.9%)	940 (72.9%)	587 (92.5%)	2 843 (80.3%)
Førde	129 (84.5%)	102 (82.4%)	176 (88.6%)	164 (87.8%)	350 (74.6%)	213 (94.8%)	1 134 (84.3%)
Bergen	445 (77.1%)	362 (74.9%)	625 (79.7%)	581 (80.6%)	1 181 (63.0%)	865 (94.2%)	4 059 (77.3%)
Fonna	224 (82.1%)	161 (83.2%)	286 (89.9%)	252 (88.1%)	619 (50.9%)	387 (93.3%)	1 929 (76.4%)
Stavanger	301 (87.0%)	268 (83.6%)	416 (79.3%)	363 (78.2%)	1 096 (53.2%)	628 (87.4%)	3 072 (72.7%)
Østfold	330 (71.2%)	321 (71.3%)	445 (80.7%)	447 (75.8%)	1 237 (65.7%)	665 (95.0%)	3 445 (75.7%)
Akershus	441 (85.5%)	438 (84.5%)	603 (88.2%)	574 (85.9%)	1 450 (73.9%)	1 102 (95.6%)	4 608 (84.5%)
OUS	136 (73.5%)	173 (76.9%)	233 (82.8%)	245 (78.4%)	511 (67.1%)	512 (95.5%)	1 810 (80.1%)
Lovisenberg	87 (83.9%)	65 (84.6%)	82 (75.6%)	84 (82.1%)	173 (71.1%)	202 (93.6%)	693 (82.4%)
Diakonhjemmet	72 (81.9%)	92 (82.6%)	143 (81.8%)	151 (78.1%)	345 (54.2%)	327 (95.4%)	1 130 (76.9%)
Innlandet	437 (77.1%)	399 (78.7%)	626 (82.6%)	547 (81.2%)	1 344 (54.7%)	897 (91.3%)	4 250 (74.5%)
Vestre Viken	413 (69.0%)	393 (69.0%)	634 (69.4%)	609 (67.2%)	1 471 (63.0%)	1 070 (94.2%)	4 590 (72.7%)
Vestfold	244 (84.8%)	254 (84.3%)	329 (79.0%)	328 (81.7%)	753 (66.7%)	538 (94.2%)	2 446 (80.0%)
Telemark	198 (73.2%)	179 (67.6%)	218 (75.2%)	232 (75.4%)	599 (68.9%)	415 (89.9%)	1 841 (75.6%)
Sørlandet	375 (79.5%)	322 (78.9%)	329 (86.6%)	347 (81.8%)	987 (69.6%)	620 (89.8%)	2 980 (79.4%)

Table B2: Associations between hospital referral area and the odds ratios of being diagnosed without cancer among CPP patients and the odds ratio for being included in CPP among cancer patients. Norway, 2015-2017. Analyses with mutual adjustment for all variables included; age, income, education, comorbidity, travel time and hospital referral area.

	Lung		Colorectal		Prostate	Breast
	Male	Female	Male	Female		
	**CPP patients: odds ratio of being diagnosed without cancer** OR (95% CI)					
**Hospital referral area**						
Finnmark	0.70 (0.48-1.02)	0.92 (0.62-1.38)	1.41 (1.04-1.92)	1.44 (1.03-2.01)	1.20 (0.92-1.56)	0.70 (0.53-0.92)
UNN	0.57 (0.43-0.76)	0.68 (0.50-0.93)	0.88 (0.71-1.08)	0.74 (0.60-0.92)	0.79 (0.66-0.94)	0.50 (0.43-0.60)
Nordland	0.65 (0.48-0.88)	0.48 (0.33-0.69)	1.22 (0.99-1.50)	1.27 (1.02-1.57)	1.05 (0.83-1.34)	0.39 (0.31-0.48)
Helgeland	1.08 (0.74-1.57)	1.29 (0.89-1.89)	1.99 (1.57-2.53)	2.24 (1.74-2.88)	1.74 (1.40-2.15)	0.45 (0.35-0.59)
Nord-Trøndelag	0.76 (0.55-1.05)	0.61 (0.43-0.87)	0.40 (0.31-0.52)	0.49 (0.38-0.64)	1.17 (0.98-1.41)	0.30 (0.24-0.38)
St. Olav	1.70 (1.35-2.13)	1.58 (1.25-2.01)	0.51 (0.42-0.62)	0.54 (0.44-0.66)	1.02 (0.88-1.18)	0.42 (0.36-0.49)
Møre og Romsdal	0.51 (0.39-0.67)	0.52 (0.38-0.71)	0.72 (0.61-0.86)	0.75 (0.62-0.89)	1.19 (1.04-1.37)	0.48 (0.41-0.55)
Førde	0.56 (0.40-0.79)	0.62 (0.42-0.92)	0.67 (0.53-0.85)	0.65 (0.51-0.84)	0.46 (0.36-0.58)	0.76 (0.62-0.93)
Bergen	0.48 (0.38-0.61)	0.56 (0.44-0.73)	0.38 (0.32-0.45)	0.41 (0.34-0.48)	0.87 (0.76-1.00)	0.80 (0.71-0.90)
Fonna	0.73 (0.56-0.95)	0.74 (0.54-1.00)	0.84 (0.69-1.02)	1.04 (0.85-1.26)	0.62 (0.51-0.76)	1.11 (0.96-1.29)
Stavanger	0.69 (0.54-0.86)	0.93 (0.73-1.19)	0.73 (0.61-0.88)	0.84 (0.70-1.01)	0.79 (0.68-0.92)	0.54 (0.47-0.62)
Østfold	1.18 (0.95-1.48)	1.02 (0.81-1.30)	0.77 (0.65-0.92)	0.82 (0.69-0.97)	0.86 (0.75-0.98)	0.71 (0.62-0.81)
Akershus	1.00	1.00	1.00	1.00	1.00	1.00
OUS	1.02 (0.75-1.39)	0.78 (0.58-1.06)	1.18 (0.96-1.44)	1.20 (0.98-1.47)	1.18 (0.99-1.40)	0.45 (0.39-0.53)
Lovisenberg	0.71 (0.49-1.03)	0.64 (0.41-1.00)	1.15 (0.84-1.58)	0.74 (0.54-1.01)	1.18 (0.91-1.53)	0.43 (0.35-0.55)
Diakonhjemmet	0.78 (0.53-1.16)	0.80 (0.56-1.17)	1.15 (0.90-1.48)	1.23 (0.97-1.57)	1.39 (1.13-1.72)	0.42 (0.34-0.51)
Innlandet	0.69 (0.55-0.86)	0.77 (0.61-0.97)	1.32 (1.14-1.53)	1.71 (1.47-1.99)	1.21 (1.06-1.38)	0.45 (0.39-0.51)
Vestre Viken	0.51 (0.40-0.65)	0.68 (0.53-0.86)	0.79 (0.67-0.93)	0.82 (0.69-0.96)	1.10 (0.97-1.25)	0.44 (0.39-0.50)
Vestfold	0.81 (0.64-1.04)	0.83 (0.64-1.06)	1.51 (1.26-1.80)	1.48 (1.24-1.77)	1.16 (1.00-1.35)	0.53 (0.46-0.62)
Telemark	0.79 (0.60-1.05)	0.95 (0.70-1.28)	2.40 (1.96-2.93)	2.21 (1.82-2.69)	0.97 (0.82-1.14)	0.26 (0.22-0.32)
Sørlandet	0.44 (0.34-0.56)	0.44 (0.33-0.57)	0.77 (0.64-0.92)	0.78 (0.65-0.94)	0.93 (0.81-1.07)	0.51 (0.44-0.59)
p-value	<0.001	<0.001	<0.001	<0.001	<0.001	<0.001
	**Cancer patients: odds ratio of being included in CPP** OR (95% CI)					
**Hospital referral area**						
Finnmark	1.01 (0.53-1.91)	0.91 (0.48-1.72)	0.66 (0.34-1.28)	1.21 (0.54-2.70)	0.79 (0.55-1.13)	3.61 (0.85-15.31)
UNN	1.34 (0.81-2.22)	1.32 (0.78-2.25)	0.83 (0.53-1.32)	0.88 (0.58-1.34)	0.85 (0.68-1.06)	1.49 (0.81-2.74)
Nordland	0.85 (0.52-1.39)	0.85 (0.51-1.43)	0.53 (0.35-0.80)	0.75 (0.48-1.17)	0.27 (0.21-0.34)	1.19 (0.60-2.34)
Helgeland	0.60 (0.33-1.10)	0.42 (0.24-0.72)	0.88 (0.49-1.56)	0.90 (0.51-1.59)	0.89 (0.65-1.21)	0.78 (0.37-1.63)
Nord-Trøndelag	0.70 (0.42-1.17)	1.00 (0.58-1.72)	0.59 (0.38-0.93)	0.71 (0.45-1.14)	0.55 (0.44-0.69)	0.41 (0.25-0.65)
St. Olav	0.55 (0.37-0.82)	0.56 (0.38-0.83)	0.54 (0.38-0.77)	0.64 (0.45-0.91)	0.68 (0.56-0.82)	0.23 (0.16-0.33)
Møre og Romsdal	0.48 (0.33-0.71)	0.55 (0.36-0.83)	0.91 (0.62-1.35)	0.68 (0.48-0.96)	0.90 (0.74-1.09)	0.61 (0.40-0.93)
Førde	0.91 (0.52-1.60)	0.80 (0.44-1.45)	1.02 (0.60-1.75)	1.20 (0.70-2.06)	1.01 (0.76-1.33)	0.87 (0.44-1.72)
Bergen	0.58 (0.41-0.82)	0.59 (0.41-0.85)	0.51 (0.37-0.70)	0.69 (0.51-0.95)	0.60 (0.51-0.72)	0.78 (0.52-1.17)
Fonna	0.80 (0.51-1.23)	0.95 (0.57-1.56)	1.18 (0.74-1.87)	1.23 (0.79-1.94)	0.34 (0.28-0.42)	0.68 (0.41-1.11)
Stavanger	1.13 (0.73-1.74)	1.00 (0.65-1.52)	0.49 (0.35-0.69)	0.61 (0.43-0.86)	0.36 (0.31-0.43)	0.33 (0.23-0.48)
Østfold	0.41 (0.29-0.59)	0.46 (0.32-0.66)	0.53 (0.38-0.75)	0.53 (0.38-0.73)	0.67 (0.56-0.79)	0.92 (0.58-1.45)
Akershus	1.00	1.00	1.00	1.00	1.00	1.00
OUS	0.50 (0.31-0.80)	0.62 (0.40-0.98)	0.65 (0.42-0.99)	0.59 (0.40-0.87)	0.76 (0.60-0.95)	0.97 (0.58-1.61)
Lovisenberg	0.89 (0.47-1.69)	1.04 (0.50-2.17)	0.41 (0.23-0.73)	0.79 (0.43-1.46)	0.81 (0.57-1.16)	0.68 (0.36-1.27)
Diakonhjemmet	0.75 (0.38-1.47)	0.81 (0.44-1.50)	0.53 (0.32-0.88)	0.58 (0.36-0.91)	0.43 (0.34-0.56)	0.92 (0.51-1.67)
Innlandet	0.59 (0.42-0.84)	0.68 (0.47-0.98)	0.63 (0.45-0.87)	0.73 (0.53-1.01)	0.41 (0.35-0.49)	0.52 (0.36-0.75)
Vestre Viken	0.36 (0.26-0.51)	0.40 (0.28-0.56)	0.28 (0.21-0.39)	0.34 (0.26-0.46)	0.60 (0.51-0.71)	0.76 (0.51-1.11)
Vestfold	0.98 (0.63-1.53)	1.03 (0.67-1.60)	0.49 (0.34-0.70)	0.76 (0.52-1.09)	0.75 (0.61-0.91)	0.79 (0.50-1.26)
Telemark	0.45 (0.29-0.68)	0.37 (0.24-0.56)	0.39 (0.26-0.58)	0.52 (0.35-0.76)	0.75 (0.61-0.93)	0.43 (0.28-0.66)
Sørlandet	0.67 (0.46-0.97)	0.66 (0.45-0.96)	0.84 (0.56-1.26)	0.75 (0.52-1.08)	0.78 (0.65-0.94)	0.43 (0.29-0.63)
p-value	<0.001	<0.001	<0.001	<0.001	<0.001	<0.001

Table B3: Comparison of the age-adjusted proportions of CCP patients diagnosed without cancer across the six CPP groups, and comparison of the age-adjusted proportion of cancer patients included in CPP across the six cancer groups, in the 21 hospital referral areas. Spearman's correlation coefficients (p-value).

	Lung		Colorectal		Prostate	Breast
	Male	Female	Male	Female		
	**CPP patients, proportion diagnosed without cancer,** ρ_s (p-value)					
Lung, Male	1.00	0.77 (<0.01)	0.30 (0.18)	0.40 (0.07)	0.12 (0.61)	-0.07 (0.76)
Lung, Female		1.00	0.26 (0.25)	0.42 (0.06)	0.13 (0.59)	0.02 (0.92)
Colorectal, Male			1.00	0.86 (<0.01)	0.53 (0.01)	-0.19 (0.41)
Colorectal, Female				1.00	0.45 (0.04)	-0.09 (0.70)
Prostate					1.00	-0.50 (0.02)
Breast						1.00
	**Cancer patients, proportion included in CPP,** ρ_s (p-value)					
Lung, Male	1.00	0.81 (<0.01)	0.41 (0.06)	0.55 (<0.01)	0.09 (0.69)	0.30 (0.19)
Lung, Female		1.00	0.29 (0.21)	0.65 (<0.01)	0.25 (0.27)	0.23 (0.31)
Colorectal, Male			1.00	0.65 (<0.01)	0.32 (0.16)	0.25 (0.28)
Colorectal, Female				1.00	0.42 (0.06)	0.34 (0.14)
Prostate					1.00	0.34 (0.13)
Breast						1.00
